# Endoscopic Endonasal Transplanum–Transtuberculum Approach for Pituitary Adenomas/PitNET: 25 Years of Experience

**DOI:** 10.3390/brainsci13071121

**Published:** 2023-07-24

**Authors:** Alessandro Carretta, Matteo Zoli, Federica Guaraldi, Giacomo Sollini, Arianna Rustici, Sofia Asioli, Marco Faustini-Fustini, Ernesto Pasquini, Diego Mazzatenta

**Affiliations:** 1 Department of Bio-Medical and Neuromotor Sciences (DIBINEM), University of Bologna, 40138 Bologna, Italyarianna.r87@gmail.com (A.R.); sofia.asioli3@unibo.it (S.A.); diego.mazzatenta@unibo.it (D.M.); 2 IRCCS Istituto delle Scienze Neurologiche di Bologna, Programma Neurochirurgia Ipofisi–Pituitary Unit, 40139, Bologna, Italy; federica.guaraldi@ausl.bologna.it (F.G.); marco.faustini@isnb.it (M.F.-F.); 3 ENT Unit, Bellaria Hospital, Azienda USL Bologna, 40139 Bologna, Italy; giacomo.sollini@ausl.bologna.it (G.S.); ernesto.pasquini@ausl.bologna.it (E.P.); 4 IRCCS Istituto delle Scienze Neurologiche di Bologna, Neuroradiology Unit, Ospedale Maggiore, 40139 Bologna, Italy; 5 IRCCS Istituto delle Scienze Neurologiche di Bologna, 40139 Bologna, Italy

**Keywords:** pituitary adenoma, PitNET, extended, transplanum, transtuberculum, endoscopic endonasal, outcome, complications

## Abstract

The role of the endoscopic transplanum–transtuberculum approach (ETTA) in the treatment of pituitary adenomas/PitNETs (PAs) is sparsely analyzed in the literature, and its use is still debated in the current practice. The aim of this study was to report our experience with this approach. Our institutional registry was retrospectively reviewed, and patients who underwent ETTA for a PA from 1998 to 2022 were included. Fifty-seven cases were enrolled over a time span of 25 years, corresponding to 2.4% of our entire PA caseload. Radical resection was achieved in 57.9% of cases, with re-do surgery (*p* = 0.033) and vessel encasement/engulfment (*p* < 0.001) as predictors of partial resection. CSF leak incidence stood at 8.8%, with higher BMI (*p* = 0.038) as its only significant predictor. Partial or full improvement of the visual field deficits was achieved in 73.5% of cases. No surgical mortality was observed. According to our results, ETTA for the treatment of PAs is characterized by a satisfactory surgical outcome but with greater morbidity than the conventional endoscopic approach. Therefore, it should be reserved for the few selected cases otherwise unsuitable for the endoscopic trans-sphenoidal route, representing a valid alternative and an effective complementary route for the transcranial approach for these challenging PAs.

## 1. Introduction

The introduction of the extended trans-sphenoidal transplanum/transtuberculum approach dates back to 1987, when Weiss demonstrated that this anterior expansion of the microsurgical trans-sphenoidal route would allow the surgeon to also approach suprasellar tumors, such as craniopharyngiomas, meningiomas or large pituitary adenomas/PitNET (PA) [1]. However, the lack of a panoramic vision and the restricted field granted by microscopical magnification prevented a widespread diffusion and impaired the popularity of this specific approach for decades [2,3,4,5,6,7,8].

Nevertheless, the introduction of the endoscope in trans-sphenoidal surgery has largely improved the intra-operative visualization of this approach, giving a panoramic and, at the same time, very detailed exposure of the nasal and skull base structures, overcoming the drawbacks associated with microscopic vision [9]. The endoscopic endonasal extended transplanum/transtuberculum approach (ETTA) has proved to be a valuable workhorse for the resection of suprasellar neoplasms (for instance, tuberculum sellae or planum meningiomas, suprasellar craniopharyngiomas—even with third ventricle involvement—suprasellar epidermoid/dermoid cysts or hypothalamic gliomas with a debulking or bioptic aim), which would otherwise require a transcranial approach, since they are not approachable through the standard endoscopic endonasal route [10,11,12,13,14,15]. 

A further rare and under-considered indication for an ETTA is represented by those cases of PA with an uncommon complex morphology, such as a major suprasellar or sub-frontal expansion, not manageable through a standard trans-sphenoidal approach and therefore requiring a transcranial or a combined transcranial–trans-sphenoidal approach [16,17,18,19,20,21]. Although the effectiveness of ETTA for these rare PAs has already been proposed in a few clinical series, most of them were not selectively focused on a PA population or were mainly aimed to describe the reconstruction technique [22,23,24,25,26,27,28].

The goal of this study was to analyze our surgical series of PAs operated through an ETTA in our center. The primary objective was the assessment of the extent of tumor resection (EOR) and the normalization rate of bio-chemical hypersecretion in functioning adenomas, identifying the factors predicting the EOR. The secondary aims were the determination of the complication rate of this approach, with particular attention to the risk of post-operative CSF leak, whose predictive factors were analyzed, and the visual and endocrinological outcomes of these patients. 

## 2. Materials and Methods

### 2.1. Study Design, Settings and Inclusion Criteria

Our prospectively collected database of all consecutive patients treated surgically in our institution (Programma Neurochirurgia Ipofisi—Pituitary Unit of IRCCS Istituto delle Scienze Neurologiche di Bologna, Italy) between 1998 and September 2022 was retrospectively reviewed to consider all the PAs treated through the ETTA. The inclusion criteria consisted of (1) histologically confirmed diagnosis of functioning or non-functioning PA; (2) adoption of an ETTA; (3) minimum follow-up of 6 months; (4) availability of all clinical and radiological pre- and post-operative features. Tumors primarily involving the cavernous sinus or the clival region, therefore treated with different extended endoscopic approaches, or those lacking complete data were excluded from the study.

Ethics committee approval and informed consent were waived for this study because of its retrospective observational design.

### 2.2. Patient Management and Surgical Nuances

The management of every single patient referred to our institution is discussed at a multi-disciplinary skull base board comprising neurosurgeons, rhinologists, neuroendocrinologists, neuroradiologists and neuropathologists, with specific expertise in skull base diseases. 

Following our management protocol, each patient underwent an endocrinological evaluation to determine the pre-operative pituitary functional status. In case of clinical suspicion of hormonal hypersecretion, specific stimulation/inhibition tests were performed. All patients also underwent ophthalmological assessment with visual acuity and visual field function determination. Clinical history was collected pre-operatively with specific attention given to previous medical, surgical or radiation treatments for the PA. Each patient performed a pre-operative contrast-enhanced MRI and a CT scan for the evaluation of nasal and paranasal sinuses’ anatomy. Based on the pre-operative MRI, each case was classified according to Barazi et al. to assess the morphological indication for an ETTA instead of conventional EEA (Table 1). 

Our surgical technique for ETTA has been extensively reported in previous reports, both for PAs and for other neoplasms [10,19,21,29]. In brief, the patient lies in a semi-sitted position under general anesthesia and orotracheal intubation. A lumbar drain is not routinely positioned. For normally pneumatized sphenoidal sinuses, we avoid the opening of the ethmoid, limiting the approach to a large anterior sphenoidectomy with extensive drilling of the floor of the sphenoidal sinus in order to completely expose its posterior wall up the planum sphenoidalis. Conversely, in case of presence of Onodi cells or other paranasal sinuses’ variants, a posterior ethmoidectomy is also performed to achieve the satisfactory exposure of the sellar bulge and tuberculum/planum notch. After bone removal and dura incision, tumor resection is performed with the standard bimanual microsurgical technique, starting with central debulking, possibly with ultrasonic aspiration (Sonopet^®^, Stryker Corporation, Kalamazoo, MI, USA; CUSA^®^ NXT or CUSA^®^ Clarity, Integra LifeSciences, Princeton, NJ, USA) for tumors with increased consistency, followed by its dissection from the neurovascular structures. Our paradigm of the skull base reconstruction technique experienced a substantial shift throughout the time span concerned, moving from a multi-layer reconstruction with fascia lata, fat, possibly bone or cartilage, and a graft of mucoperiosteum to a similar technique but using a dural substitute (Biodesign^®^, Cook Medical LLC, Bloomington, IN, USA) instead of fascia lata, fat, possibly bone or cartilage, covered by a naso-septal pedicled flap.

All histopathological diagnoses were retrospectively reviewed by a boarded neuropathologist (S.A.) according to the 2022 5th edition of WHO Classification of Tumors of Endocrine Organs [30]. ENT evaluation to assess the healing and remucosalization of the nasal cavity was performed one month after the procedure and as required subsequently. Bio-humoral assays, ophthalmological and neurological examinations, and post-contrast MRI were repeated 3 months after surgery and then annually.

Tumor resection was defined as radical in case of no tumor remnant at MRI at 3 months or non-radical in case a remnant tumor was detected. Clinical outcomes were evaluated based on follow-up examinations as normalized/improved, unchanged or worsened. Recurrence or tumor progression were evaluated at follow-up, as well as any following adjuvant treatment. 

**Table 1:** Morphological classification of the PAs suitable for the ETTA approach, adapted from Barazi et al. [21].

Type 1	Ectopic peduncular or supradiaphragmatic peri-infundibular PAs, including ectopic microadenomas of the pituitary stalk or purely supradiaphragmatic macroadenomas (mostly remnant or recurrence after previous partial surgeries). These tumors are not suitable for an EEA because they have no sellar infradiaphragmatic component.
Type 2	PAs with sub-frontal extension, including macroadenomas with a supra- or infradiaphragmatic sub-frontal extension. These tumors are not fully resectable with an EEA because of their sub-frontal component, which extends anteriorly with an unfavorable angle and direction for the trans-sphenoidal approach.
Type 3	PAs presenting with a major extrasellar component, including macroadenomas with suprasellar supradiaphragmatic component exceeding the sellar volume (i.e., air balloon PAs) unlikely to be delivered through the sella with an EEA, and macroadenomas with both a large intrasellar infradiaphragmatic part and a large suprasellar supradiaphragmatic portion connected through a narrow isthmus (i.e., snowman PAs), which impairs their resection through an EEA.

### 2.3. Data Sources and Variables Included

Clinical, neuroradiological and surgical data, derived from the previously described pre- and post-operative examinations, were prospectively included in a digital anonymized archive. 

The primary endpoint of the study was represented by the EOR rate, and the secondary endpoint consisted of the determination of the complication rate and patient endocrinological and visual outcomes. The following parameters were retrospectively collected and compared according to the study endpoints: (1) age; (2) BMI; (3) previous surgical or radiation treatments; (4) patient referral symptom leading to diagnosis; (5) pre-operative pituitary functional status; (6) pre-operative visual function; (7) tumor maximal diameters (measured in anteroposterior, laterolateral and craniocaudal extensions); (8) lesion volume; (8) maximal cranial extension of the lesion and morphology (up to suprasellar cistern, third ventricle or foramina of Monro); (9) tumor consistency; (10) vessel encasement/engulfment; (11) subarachnoid invasion; (12) type of surgical approach; (13) tumor morphology according to Barazi et al. (Table 1) [21]; (14) skull base reconstruction technique. The rate of CSF leak was also compared between the first and second half of this patient series to assess the impact of surgeon experience and of the introduction of the naso-septal flap for this complication.

Continuous variables are outlined as mean (±standard deviation). The qualitative radiological parameters were independently evaluated by three blinded researchers (A.C., M.Z., A.R.), and a discussion took place in case of disagreement. For the continuous data collected, the mean among the three measurements was calculated and considered for statistical purposes.

### 2.4. Statistical Analysis

Statistical univariate analysis was performed with IBM SPSS Statistics Version 29.0.0.0 (IBM Corp. Released 2022. IBM SPSS Statistics for Mac. Armonk, NY, USA: IBM Corp.). 

Normal distribution was analyzed with the Shapiro–Wilk test. According to their normal or non-normal distribution, continuous variables (age, BMI, volume, maximal diameter) were compared using Student’s *t*-test or the Mann–Whitney *U*-test. Similarly, all the other categorical variables were cataloged in contingency tables according to the analyzed outcomes and compared with a chi-squared test. Parameters with significant correlation with study outcomes based on univariate analysis were further compared with a multi-variate logistic regression.

The *p*-value was assumed to be statistically significant at ≤0.05.

## 3. Results

In the time span included, a total of 2351 endoscopic adenomectomies were performed at our institution. After extensive review and application of the inclusion and exclusion criteria, 57 (2.4% of our case series) procedures were included in this study. Thirty-five (61.4%) patients were male, and the mean age was 54.1 ± 13.5 years. 

Twenty-six (45.6%) patients were naïve for previous surgical or radiation treatments, while, as reported in Table 2, among the thirty-one patients already operated, one of them also underwent external-beam radiotherapy. The most common symptoms leading to diagnosis were visual deficits (32, 56.1%), followed by endocrinological disturbances (15, 26.3%). Of note, in six (10.5%) cases, the tumor was an incidental finding. 

Most PAs were non-functioning (43, 75.4%), and the remaining PAs included five PRL-secreting adenomas, four ACTH-secreting adenomas, four GH-secreting adenomas and one TSH-secreting adenoma. Pre-operative endocrinological hypopituitarism was observed in 17 cases (29.8%) and DI in 2 cases (3.5%). Pre-operative visual acuity deficits were present in 6 cases (10.5%) and field deficits in 34 cases (59.6%). The mean maximal tumor diameter was 30.3 ± 12.8 mm, and the mean tumor volume was 11.3 cm^3^ ± 16.8. Their cranial extension reached the suprasellar cistern in 24 cases (42.1%), third ventricle in 22 cases (38.6%) and foramina of Monro in 11 cases (19.3%). According to Barazi et al., they can be classified as type 1 in 16 cases (28.1%), as type 2 in 7 cases (12.3%) and as type 3 in 34 cases (59.7%). Intra-operatively, 12 tumors (21.1%) were firm; vascular encasement/engulfment was observed in 9 cases (15.8%) and subarachnoid infiltration in 37 cases (64.9%) (Table 3). In 22 cases (38.6%), closure was performed with fascia lata, fat, possibly bone or cartilage, and mucoperiosteal graft. In 32 cases (56.1%), closure was performed with Biodesign, fat, possibly bone or cartilage, and a naso-septal flap.

Radical resection was achieved in 33 cases (57.9%), and hypersecretion was resolved in 9 cases (64.3%) (Table 4). All cases of functioning PAs not in remission after surgery only underwent a specific medical therapy, and in four cases, also irradiation (either with conventional radiotherapy, radiosurgery or adrotherapy), bringing the hypersecretion under control in all cases at follow-up. The complications consisted of one case (1.8%) of post-operative epistaxis, one case (1.8%) of meningitis requiring antibiotic treatment, two cases (3.5%) of silent lacunal ischemia of the head of caudate nucleus or the temporal pole revealed by post-operative imaging, two (3.5%) cases of transient third cranial nerve palsy (resolved at discharge), one case (1.8%) of transient diabetes insipidus (DI) resolved at discharge, six (10.5%) cases of surgical field hematomas treated conservatively in three cases and requiring surgical treatment in the other three (two through an EEA and one through a TCA). Among those, one patient (1.7.%) experienced hydrocephalus, requiring ventricular-peritoneal shunt. One patient (1.7%) with severe cardiovascular comorbidities developed a multiple-organ disfunction syndrome not related to surgical complications and passed away in ICU one month after the procedure. No cases of mortality due to surgical complications were reported. 

Visual acuity and field deficits resolved or improved, respectively, in 2 (33.3%) and 25 (73.5%) cases, while post-operative visual acuity or field worsening was demonstrated in 2 cases each (Table 5). Conversely, a worsening of the anterior pituitary function was observed in 17 (29.8%) cases, and 15 (26.3%) patients developed a DI. 

At follow-up (mean 42.5 ± 30.7 months), three cases (5.2%) presented a recurrence after GTR, and they were treated, respectively, with radiosurgery in one case, transcranial resection in another case and further endoscopic endonasal resection in the latter case. Progression of a remnant tumor was reported in six (10.5%) cases, which was treated with irradiation in five cases (two with conventional radiotherapy and three with radiosurgery) and further standard endoscopic endonasal resection in one case. Alongside the aforementioned patient who died from cardiovascular comorbidities in the ICU, another one died from unrelated causes during follow-up. All the other patients included (96.5%) were alive at follow-up.

### Statistical Analysis

According to the univariate analysis, a partial EOR correlates with previous surgeries (*p* = 0.033) and the presence of vessel encasement/engulfment (*p* < 0.001).

As reported in Table 6, higher BMI was the only parameter reported to be significantly correlated with post-operative CSF leak after ETTA (*p* = 0.038). CSF leak incidence was not reported to be significantly correlated with the skull base reconstruction technique (*p* = 0.647 and *p* = 0.618) or to exhibit differences (*p* = 0.669) between the first half of the patients treated with the ETTA approach (7.14%) and the second half (10.34%). 

Post-operative anterior hypopituitarism correlates with male sex (*p* = 0.034) and previous surgeries (*p* = 0.049). Conversely, the development of post-operative DI correlates with higher tumor volume (*p* = 0.048) and maximal diameter (*p* = 0.006), vessel encasement/engulfment (*p* = 0.030), subarachnoid invasion (*p* = 0.040) and inclusion of the simultaneous transcranial approach (*p* = 0.016). 

An unsatisfactory visual acuity outcome, with post-operative worsening or absence of any improvement in pre-operative deficits, correlates with higher tumor volume (*p* = 0.027) and maximal diameter (*p* = 0.017). Similarly, an unsatisfactory visual field outcome is reported to be linked to previous surgeries (*p* = 0.024).

The multi-variate analysis performed by means of a logistic regression did not disclose any significant correlation.

## 4. Discussion

In our study, we demonstrated on a large series of 57 patients with PAs operated through an ETTA that this approach can both represent a valid alternative and an effective complementary route for the TCA, with a radical resection rate of 57.9% (33 cases) and hypersecretion resolution in 64.3% of cases (9 out of 14). Indeed, the ETTA has proved not only to allow the surgeon to manage through a trans-sphenoidal route those adenomas not approachable with a standard EEA and otherwise requiring a TCA (for example, because of their purely supradiaphragmatic location or due to atypical irregular morphology); it was also proved that the ETTA can be combined with a TCA, as reported in two cases in our series, for those asymmetrical tumors whose lateral extension would represent a limit for the ETTA. This is, to our knowledge, the largest surgical case series focusing on ETTA, which includes and compares all the different types of PAs suitable for the approach and discusses their indications. 

### 4.1. Classification, Surgical Indications and EOR

**Figure 1:**
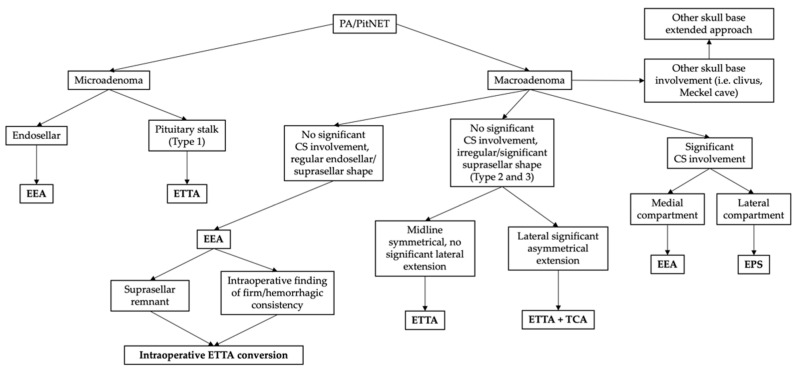
Illustrative flow chart of our general decisional algorithm for PA surgical management. Each approach should be tailored to the clinical–radiological features and intra-operative findings of the individual case. CS: cavernous sinus; EEA: standard endoscopic endonasal approach; EPS: ethmoidopterygosphenoidal approach [33]; ETTA: endoscopic endonasal transplanum–transtuberculum approach; TCA: transcranial approach.

The indications for an ETTA for PAs have been controversial in clinical practice and in the dedicated literature, and few studies have specifically considered this topic [16,17,20,21]. While well established in clinical practice for complex anterior skull base neoplasms, the ETTA conflicts with some fundamental principles of pituitary surgery, such as avoidance of diaphragm violation, with the consequent intra-operative CSF leak, and selective tumor resection, with the sparing of the gland and stalk structures to preserve the endocrinological function. Moreover, in the vast majority of cases, PAs—also including large or giant tumors—arise inside the sella from the pituitary gland, and they usually extend toward the suprasellar space in a caudocranial direction, displacing the diaphragm upwards. Therefore, after central debulking of the intrasellar part, the dome progressively descends in a downward direction, increasing its likelihood of being delivered through a conventional endoscopic endonasal approach without any need for a supradiaphragmatic extension. It has been hypothesized that less than 10% of PAs have an unsuitable morphology for a conventional EEA, thus requiring an alternative route, such as the TCA [31,32]. In 2013, Barazi et al. proposed that for these rare and selected cases unsuitable for a standard EEA, the ETTA could be considered as an alternative to the TCA, combining the advantages of the trans-sphenoidal corridor with the possibility to resect supradiaphragmatic PAs (Figure 1). Our series confirms that only few cases require this extended approach, accounting for 2.4% in our series of 2351 endoscopic endonasal adenomectomies, which should be considered exclusively for those cases with peculiar features, which makes them unapproachable with an EEA, which remains the first choice for PAs. In particular, based on the tumor location and morphology, these authors identify three possible types of PAs potentially suitable for the ETTA. 

**Figure 2:**
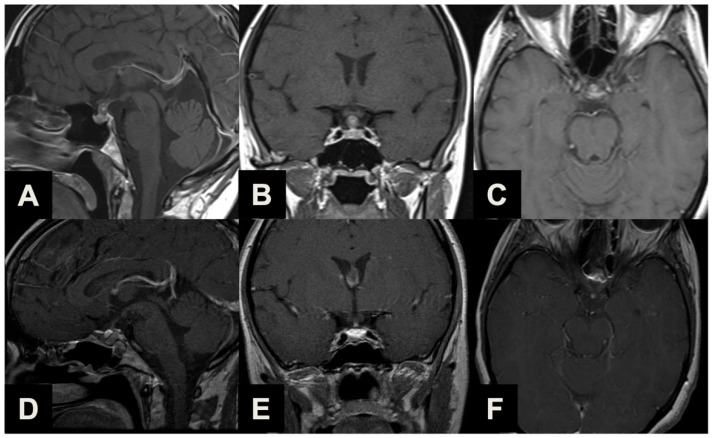
Illustrative case of a Type 1 sec. Barazi PA suitable for ETTA. (**A**–**C**) Midsagittal (**A**), coronal (**B**) and axial (**C**) pre-operative contrast-enhanced T1-weighted MR images of a 35-year-old female complaining of visual field disturbances, with a clinically manifest bitemporal hemianopsia. Imaging and laboratory exams reported a non-functioning supradiaphragmatic macroadenoma along the pituitary stalk, slightly compressing the optic chiasm. She underwent ETTA, which achieved radical resection with an unremarkable clinical course and a resolution of pre-operative symptomatology. (**D**–**F**) Midsagittal (**D**), coronal (**E**) and axial (**F**) post-operative contrast-enhanced T1-weighted MR images.

Ectopic peduncular or supradiaphragmatic peri-stalk PAs (Type 1, Table 1, Figure 2) are uncommon occurrences, as represented in our series (16, 28.1%), mostly including 5 (31.3%) ectopic secreting microadenomas of the pituitary stalk and 11 (68.7%) remnants or recurrences after previous partial surgeries of purely supradiaphragmatic macroadenomas. These tumors are not suitable for a conventional EEA because of the lack of any sellar infradiaphragmatic component, thus requiring a complete supradiaphragmatic corridor. 

**Figure 3:**
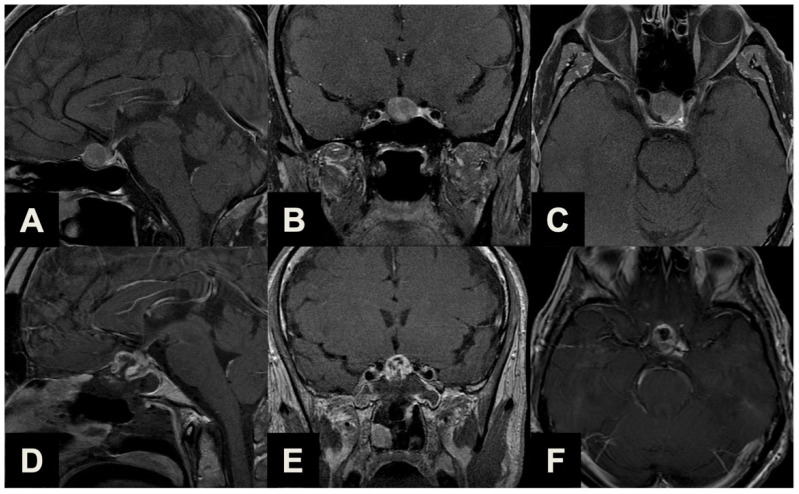
Illustrative case of a Type 2 sec. Barazi PA suitable for ETTA. (**A**–**C**) Midsagittal (**A**), coronal (**B**) and axial (**C**) pre-operative contrast-enhanced T1-weighted MR images of a 63-year-old male patient previously treated with a standard endoscopic endonasal approach for a non-functioning pituitary macroadenoma. Years later, a linearly progressing supradiaphragmatic recurrence with sub-frontal extension was observed. He underwent ETTA, which achieved near-radical resection (a small remnant was revealed with post-operative imaging posteriorly) with an unremarkable clinical course. (**D**–**F**) Midsagittal (**D**), coronal (**E**) and axial (**F**) post-operative contrast-enhanced T1-weighted MR images.

PAs with sub-frontal extension (Type 2, Table 1, Figure 3) are rare tumors (7, 12.3%) with a supra- or infradiaphragmatic sub-frontal extension, which extends rostrally up or beyond the tuberculum sellae, which prevents this portion from descending into the intrasellar cavity during tumor resection and which would require an unfavorable approach direction for a conventional EEA. 

**Figure 4:**
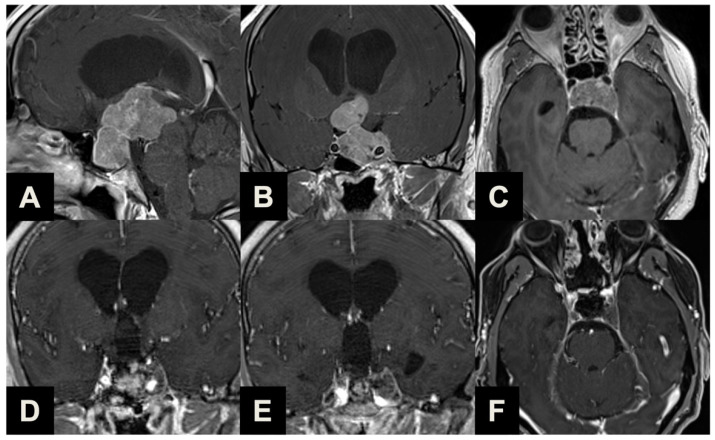
Illustrative case of a Type 3 sec. Barazi PA suitable for ETTA. (**A**–**C**) Midsagittal (**A**), coronal (**B**) and axial (**C**) pre-operative contrast-enhanced T1-weighted MR images of a 40-year-old male patient complaining of cognitive decline and urinary incontinence. Imaging and laboratory exams reported a non-functioning macroadenoma with a significant suprasellar portion, obliterating the third ventricle and causing hydrocephalus (Type 3). Visual examination revealed bitemporal hemianopsia. He underwent ETTA, which achieved near-radical resection (a small remnant was revealed with post-operative imaging near the left cavernous sinus). The patient experienced severe panhypopituitarism and diabetes insipidus, which required persistent complete substitution therapy; conversely, a complete resolution of pre-operative visual field deficit, as well as cognitive and urinary symptomatology was observed. (**D**–**F**) Coronal (**D**,**E**) and axial (**F**) post-operative contrast-enhanced T1-weighted MR images.

Finally, the most common indication for an ETTA was represented in our series by PAs presenting with a major extrasellar component (34, 59.6%) (Type 3, Table 1, Figure 4), such as macroadenomas with a suprasellar supradiaphragmatic component exceeding the sellar volume (i.e., air balloon PAs) unlikely to be delivered through the sella with an EEA, and macroadenomas with both a large intrasellar infradiaphragmatic part and a large suprasellar supradiaphragmatic portion connected through a narrow isthmus (i.e., snowman PAs), which impairs their resection through an EEA. 

In some cases, a firm consistency may prevent the dome of the tumor from descending into the surgical cavity, and we primarily prefer to avoid extending the approach to these cases, instead using angled instruments and scopes to entirely remove the tumor. However, we noted that an increased consistency was reported in a significant number of PAs (21.1%), confirming that these tumors represent a challenge for the pituitary surgeon.

The flexibility of the approach, developed as an extension of the standard endoscopic endonasal route, also provides an opportunity for intra-operative conversion if required by the neoplasm features and surgical findings. Although in many cases, an ETTA is planned from the very beginning of the procedure (purely supradiaphragmatic neoplasms, major suprasellar extension), in other cases, where the standard endoscopic endonasal approach fails to achieve a satisfactory result (i.e., firm consistency of the neoplasm, non-descending diaphragm leading to suprasellar remnants), it can be intra-operatively extended to the ETTA to gain access to the supradiaphragmatic space. It is therefore our standard practice to preserve the septal mucosa during the first steps of every PA resection procedure, in case the harvesting of the naso-septal flap would later be unexpectedly necessary.

Radical resection was achieved in 57.9% of cases, in line with previous reports [17]. This result should be outlined in the context of a highly complex case series, encompassing very large lesions with atypical morphology, vessel encasement and a significant number of secondary treatments. Indeed, comparing these results with those reported for the TCA series, we can observe a comparable degree of resection [32,34,35,36]. In our study, surgery for remnants and recurrences was observed to be a predictor of PR (*p* = 0.033), probably due to the presence of adherences and scarring from previous approaches, precluding optimal and safe surgical maneuvers [37]. Moreover, a close vessel relationship was also a factor precluding GTR (*p* < 0.001), considering that surgical dissection around major cranial vessels is extremely challenging, even for experienced hands [38]. It is also conceivable that advanced intra-operative imaging tools, such as intra-operative MRI, although never used in our surgical series, could help the surgeon increase the EOR by means of locating small remnants in the surgical cavity not detected by the surgeon’s eye, as reported in the literature [39,40]. Conversely, this could lengthen the duration of the procedure.

**Figure 5:**
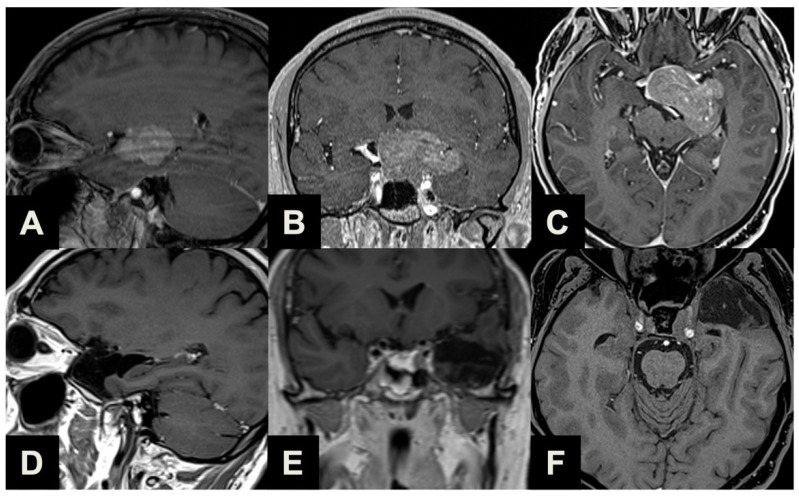
Illustrative case of a large PA suitable for combined ETTA–TCA. (**A**–**C**) Parasagittal (**A**), coronal (**B**) and axial (**C**) pre-operative contrast-enhanced T1-weighted MR images of a 55-year-old male patient complaining of visual disturbances, with a clinically manifest bitemporal hemianopsia and left eye visual impairment consistent with second left cranial nerve involvement. Imaging and laboratory exams reported a non-functioning macroadenoma with a significant left lateral extension, invading and obliterating the ipsilateral basal cisterns. He underwent ETTA with a left TCA in the same surgical session, which achieved near-radical resection. Two millimetric remnants were revealed with post-operative imaging at the level of left cavernous sinus and interpeduncular cistern. The patient experienced a clinically silent left temporal pole ischemia, severe post-operative panhypopituitarism and diabetes insipidus, which required persistent complete substitution therapy; conversely, a complete resolution of pre-operative visual acuity and field deficits was observed. (**D**–**F**) Parasagittal (**D**), coronal (**E**) and axial (**F**) post-operative contrast-enhanced T1-weighted MR images.

Although the ETTA represents an excellent extracranial approach for the suprasellar space, avoiding any brain retraction or vasculo-nervous manipulation, it poses an intrinsic drawback for the more lateral tumor extension than the carotid and the optic nerve planes. In these cases, the TCA could be proposed as a complementary approach to the ETTA (Figure 5) [41]. The lateral growing pattern of the neoplasm usually provides a “natural” surgical corridor for the TCA, while the ETTA helps the surgeon resect and debulk the median and paramedian part of the lesion, addressing the deepest intra- and suprasellar portion abutting the chiasm or the third ventricle from the ventral corridor, allowing for the achievement of a greater EOR and reducing the risk of recurrence [42]. However, it should be remarked that in our series, we noted that the combination of an ETTA with a TCA would add significant morbidity, namely increasing the risk of post-operative DI (*p* = 0.018) [43]. Therefore, the choice of a combined TCA–ETTA approach should be balanced in an optimal risk–benefit assessment.

### 4.2. Surgical Complications

The incidence of post-operative CSF leak in our case series was 8.7% (five cases). All of them underwent a prompt endoscopic endonasal revision, and none of them developed meningitis. These results are in line with those reported by Khan et al., who assessed a pooled CSF leak incidence of 9% in a recent systematic review of extended endonasal approaches [44]. Indubitably, this rate is higher than those reported for conventional EEA, and this represents the main disadvantage of this approach. Throughout the years, the developing surgical experience and skills and the introduction of innovative repair techniques for large osteo-dural defects, such as the naso-septal flap, have led to an evolution in our techniques for skull base reconstruction. In our series, we observed no significant difference in CSF leak rates when comparing patients who underwent reconstruction with different techniques, as well as between the first and the second half of the case series. The only predictor of CSF leak was represented by higher BMI, which was also recognized as a negative prognostic factor in other series of endoscopic endonasal skull base cases [45,46,47]. Moreover, a careful management of post-operative nasal care, with periodic saline irrigation and ENT evaluations, allows the patients to preserve an acceptable quality of life during the uncomfortable phase of crusting and nasal remucosalization, quantified over three months [21,48,49].

Other complications were represented by the following incidences of 1.8% of post-operative epistaxis, 1.8% of meningitis requiring antibiotic treatment, 3.5% of asymptomatic brain ischemia, 3.5% of transient third cranial nerve palsy and 10.5% of surgical field hematomas, which required surgical treatment in 50% of cases. Analyzing the non-negligible incidence of surgical field hematomas, the remnant tumor apoplexy could be hypothesized as a strong risk factor for this occurrence. Unexpectedly, in only three cases (out of a total of six experiencing post-operative hemorrhages) was a non-radical resection performed. It is our opinion that, despite tumor remnant apoplexy being a crucial issue very well known to pituitary surgeons, the suprasellar, subarachnoid extension of our approach could slightly increase per se the risk factor for post-operative bleeding, unavoidably manipulating small capillaries and branches of hypophyseal arteries, which could bleed in a large emptied post-operative surgical cavity. Conversely, a resection as extensive as permitted, respecting vascular anatomy, should be the primary concern for the pituitary surgeon to also decrease the occurrence of swelling and bleeding of the remnant (especially in those cases, where a gross total resection is not amenable), whose risk, although reducible, could never be zero. The incidence of these complications represents a consequence of the expansion of the surgical approach into the supradiaphragmatic space. Comparing our complication rates with other ETTA series, we observe a similar incidence [21,50,51], and it is important to remark that they are in line with the overall complication rates of the TCA approaches, confirming that, although the ETTA has a higher complication rate compared to the EAA, it is not more unfavorable than the TCA [32]. 

### 4.3. Clinical Outcome

The most significant advantage of the ETTA is represented by the favorable clinical outcome. Full or partial regression of pre-operative visual acuity and field symptoms was observed in, respectively, 33.3% and 73.5% of cases. As reported by many authors, early decompression of the optic structures and vessel-preserving dissection enabled by the ETTA are the key features determining such positive results, which are significantly superior to the TCA for sellar and suprasellar pathologies [50,52,53]. Similarly, our endocrinological outcome, with 26.3% of new-onset DI and 29.8% of anterior pituitary function worsening, seems not to be inferior to the TCA [54]. 

In the context of an optimal multi-disciplinary management, the individuation of the predictors of visual and endocrinological post-operative impairment is crucial. In our cohort, the major predictors of post-operative DI were the volume (*p* = 0.048), maximal diameter (*p* = 0.006), vessel encasement (*p* = 0.030), subarachnoid invasion (*p* = 0.040) and inclusion of the TCA (*p* = 0.016). Neurohypophysis and pituitary stalk are delicate structures, which are strongly affected when performing the ETTA. If in smaller lesions, namely Type 1 and 2, the stalk can be visualized, dissected and preserved early, in larger invasive lesions, it is displaced, and it can be inadvertently harmed after surgical maneuvers and dissection [55]. Similarly, lesions with vessel encasement and subarachnoid invasion require prolonged surgical maneuvering, increasing the risk of meningo-hypophysial artery damage with consequent pituitary function deficits. Similarly, the neurovascular manipulation and dissection unavoidable in transcranial approaches represents a negative prognostic predictor of post-operative DI development. The predictors of unsatisfactory visual outcomes were larger lesion volume (*p* = 0.027), diameter (*p* = 0.017) and re-do surgery (*p* = 0.024) for comparable reasons with the involvement of optic nerves, chiasm and tracts. 

### 4.4. Strengths and Limitations

The main strength of this study is that all the patients were treated and managed in the same referral center by a highly specialized multi-disciplinary team according to the same established surgical core principles (with expected improvements and implementations throughout the years), with adequate homogeneity. Moreover, despite its observational retrospective design, no patients were excluded from the study for lacking essential data. Conversely, the cohort size (even in a large referral center for pituitary neoplasms) precludes us from performing an effective advanced statistical analysis, such as a multi-variate analysis, limiting the generalizability of our findings, especially in a heterogenous cohort. Moreover, a thorough analysis of the post-operative endocrinological management of hypersecreting adenomas, progression-free survival for remnants and adjuvant treatments is beyond the scope of this paper.

## 5. Conclusions

In our study, we observed and highlighted in a large cohort that the ETTA can also play a significant role in PAs surgery, and it should be part of the pituitary surgeons’ armamentarium. Indeed, it has both an alternative and complementary role with the TCA, expanding the indications of the endoscopic trans-sphenoidal approach to complex PAs unsuitable for the EEA, and it can also be combined with a TCA for tumors with significant lateral extension. Without the ETTA, the only surgical option for the management of those PAs would have been the transcranial route—either standalone or combined with a standard endoscopic endonasal approach—with major invasivity.

However, it should be considered that the ETTA presents a higher rate of complications—particularly the post-operative CSF leak—than the standard EEA, suggesting that it should be reserved for the few selected cases with strict indications. For these tumors, such morbidity is balanced by the advantages afforded by the EEA, with a favorable patient outcome and with particularly satisfactory visual and endocrinological results.

The identification of pre-operative factors predicting complications and unsuccessful outcomes (mainly re-do surgeries for recurrences, larger lesions, higher BMI, subarachnoid invasion and strict vessel relationship) is crucial for providing an accurate patient-tailored treatment and an optimal post-operative management. Further, multi-centric studies are warranted for a better characterization of those features. 

## Figures and Tables

**Table 2:** Pre-operative demographic, clinical and radiological features of the included cohort. EEA: extended endoscopic endonasal approach; EA: endoscopic endonasal approach; TCA: transcranial approach; DI: diabetes insipidus.

		N, % or SD
Sex	Male	35, 61.4
	Female	22, 38.6
Age		54.1 ± 13.5
BMI		27.8 ± 5.4
Previous treatment	Naïve	26, 45.6
	Previous surgery	30, 52.6
	Previous surgery and radiotherapy	1, 1.8
Previous surgical procedure	EEA	20, 64.5
	EA + TCA	2, 6.5
	TCA	5, 16.1
	Microsurgical trans-sphenoidal	4, 12.9
First clinical manifestation	Cognitive decline	3, 5.3
	Endocrinological hyperproduction	15, 26.3
	Visual disturbances	32, 56.1
	Incidental	6, 10.5
	Headache	1, 1.8
Endocrinological hypersecretion at admission	PRL	5, 8.8
	ACTH	4, 7
	GH	4, 7
	TSH	1, 1.8
	No	43, 75.4
Endocrinological impairment at admission	Anterior partial hypopituitarism	1, 1.8
	Anterior panhypopituitarism	14, 24.6
	DI	0, 0
	Anterior partial hypopituitarism and DI	1, 1.8
	Panhypopituitarism and DI	1, 1.8
	No	40, 70.2
Visual acuity impairment at admission	Yes	6, 10.5
	No	51, 89.5
Visual field impairment at admission	Yes	34, 59.6
	No	23, 40.4
Volume, cm^3^		13.8 ± 16.8
Maximal diameter, mm		30.3 ± 12.8
Cranial extension	Foramina of Monro	11, 19.3
	Third ventricle	22, 38.6
	Suprasellar cistern	24, 42.1
Morphology according to Barazi et al. [21]	Type 1	16, 28.1
	Type 2	7, 12.3
	Type 3	34, 59.6

**Table 3:** Intra-operative surgical findings and features of the included cohort.

		N, %
Consistency	Soft	45, 78.9
	Firm	12, 21.1
Vessel encasement/engulfment	Yes	9, 15.8
	No	48, 84.2
Subarachnoid invasion	Yes	37, 64.9
	No	20, 35.1
Type of surgical approach	ETTA	55, 96.5
	ETTA + TCA (one step)	2, 3.5
Closure with fascia lata, fat, possibly bone and cartilage, and mucoperiosteal graft	Yes	22, 38.6
	No	35, 61.4
Closure with Biodesign, fat, possibly bone and cartilage, and naso-septal flap	Yes	32, 56.1
	No	25, 43.8

**Table 4:** Surgical outcome, hypersecretion resolution and complications observed in the included cohort.

		N, %
EOR	Radical	33, 57.9
	Non-radical	24, 42.1
Hypersecretion post-operative normalization	PRL	3, 60
	ACTH	4, 100
	GH	1, 25
	TSH	1, 100
	Residual hypersecretion	5, 35.7
Complications	CSF leak	5, 8.8
	Epistaxis	1, 1.8
	Meningitis	1, 1.8
	Asymptomatic brain ischemia	2, 3.5
	Transient third cranial nerve palsy	2, 3.5
	Transient DI	1, 1.8
	Hematoma	6, 10.5
	Hydrocephalus requiring VPS	1, 1.8

**Table 5:** Endocrinological and visual clinical outcomes of the included cohort.

		Normalized/Improved	Unchanged	Worsened
		N, %	N, %	N, %
Endocrinological disturbances	Intact	0, 0	22, 55	18, 45
	Anterior partial hypopituitarism	0, 0	0, 0	1, 100
	Anterior panhypopituitarism	0, 0	10, 71.4	4, 28.6
	DI	0, 0	0, 0	0, 0
	Anterior partial hypopituitarism and DI	0, 0	1, 100	0, 0
	Panhypopituitarism and DI	0, 0	0, 0	1, 100
Visual acuity deficits	Intact	0, 0	49, 86	2, 4
	Present	2, 33.3	4, 66.7	0, 0
Visual field deficits	Intact	0, 0	23, 100	0, 0
	Present	25, 73.5	7, 20.6	2, 5.9

**Table 6:** Univariate analysis showing correlations between the parameters included and study outcomes (*p* values).

Parameter	EOR	CSF Leak	New-Onset Anterior Pituitary Impairment	New-Onset DI	Visual Acuity Outcome	Visual Field Outcome
BMI *	0.177	***0.038***	0.307	0.314	0.089	0.220
Age ^§^	0.615	0.331	0.235	0.659	0.366	0.467
Sex	0.885	0.647	***0.034***	0.269	0.780	0.695
Volume *	0.482	0.593	0.092	***0.048***	***0.027***	0.827
Maximal diameter ^§^	0.188	0.301	0.128	***0.006***	***0.017***	0.387
Remnant/recurrence	***0.033***	0.362	***0.049***	0.057	0.523	***0.024***
Consistency	0.972	0.719	0.067	0.534	0.781	0.061
Vessel encasement	***<0.001***	0.591	0.802	***0.030***	0.213	0.116
Subarachnoid invasion	0.174	0.332	0.558	***0.040***	0.924	0.904
TCA	0.091	0.655	0.525	***0.016***	0.064	0.177
Third ventricle extension	0.116	0.385	0.206	0.423	0.645	0.373
Morphology according to Barazi et al. [21]	0.906	0.622	0.178	0.328	0.104	0.428
Closure with fascia lata	-	0.647	-	-	-	-
Closure with naso-septal flap	-	0.618	-	-	-	-

*: Mann–Whitney *U*-test. ^§^: Student’s *t*-test.

## Data Availability

The authors declare that the gathered data included and used for the analysis outline are available in the manuscript. Further datasets are available upon reasonable request from the corresponding author.
